# Clinical effectiveness and clinical toxicity associated with platinum-based doublets in the first-line setting for advanced non-squamous non-small cell lung cancer in Chinese patients: a retrospective cohort study

**DOI:** 10.1186/1471-2407-14-940

**Published:** 2014-12-12

**Authors:** Yan Wang, Jianhua Chen, Shengqi Wu, Chenping Hu, Xiaoling Li, Yuqin Wang, Yicheng Yang, Narayan Rajan, Yun Chen, Yi Chen, Zhuanzhuan Luo, Wendong Chen

**Affiliations:** Department of Medical Oncology, Cancer Institute (Hospital), Chinese Academy of Medical Sciences and Peking Union Medical College, Beijing, China; Department of Medical Oncology, Hunan Province Tumor Hospital, Central South University, Changsha, Hunan China; Department of Research and Education, Hunan Province Tumor Hospital, Central South University, Changsha, Hunan China; Department of Respiratory, Xiangya Hospital, Central South University, Changsha, Hunan China; Department of Pharmacy, Xuanwu Hospital, Capital Medical University, Beijing, China; Lilly Suzhou Pharmaceutical Co., Ltd, Shanghai Branch, Shanghai, China; Global Patient Outcomes and Real World Evidence, Eli Lilly, Indianapolis, IN USA; Division of Health Outcome Research, Normin Health Changsha Representative Office, Changsha, Hunan China; Division of Social and Administrative Pharmacy, Leslie Dan Faculty of Pharmacy, University of Toronto, Toronto, Ontario Canada; Normin Health, Toronto, Ontario Canada

**Keywords:** Non-squamous NSCLC, First-line, Chemotherapy, Treatment failure, Adverse events, Pemetrexed

## Abstract

**Background:**

Real-world evidence lacks for clinical effectiveness and clinical toxicity associated with platinum-based doublets in the first-line setting for advanced non-squamous non-small cell lung cancer (advNS-NSCLC) in Chinese patients.

**Methods:**

Patients receiving first-line chemotherapy for advNS-NSCLC in four Chinese tertiary care hospitals from 2007 to 2012 were retrospectively identified for chart review. Propensity score methods created best matched pairs for platinum/pemetrexed versus other platinum-based doublets for head-to-head comparisons of early treatment discontinuation (completed treatment cycles <4), treatment failure (progressive disease or early treatment discontinuation), and adverse events (AE). Conventional multiple logistic regression analyses were also performed to confirm the impact of the studied platinum-based doublets on early treatment discontinuation, treatment failure, and hematological AE using vinorelbine/platinum as reference.

**Results:**

1,846 patients were included to create propensity score matched treatment groups for platinum/pemetrexed versus docetaxel (95 pairs), paclitaxel (118 pairs), gemcitabine (199 pairs), and vinorelbine (72 pairs)-contained doublet, respectively. Platinum/pemetrexed was associated with significantly lower risks of early treatment discontinuation (odds ratio (OR) ranged from 0.239, p = 0.001 relative to platinum/docetaxel to 0.389, p = 0.003 relative to platinum/paclitaxel) and treatment failure (OR ranged from 0.257, p < 0.001 relative to platinum/paclitaxel to 0.381, p < 0.001 relative to platinum/gemcitabine) than the other four studied doublets. Platinum/pemetrexed was also associated with several significantly lower hematological AE rates, such as versus platinum/paclitaxel (any hematological AE: OR 0.508, p = 0.032), platinum/gemcitabine (i.e., any hematological AE: OR 0.383, p < 0.001; anemia: OR 0.357, p < 0.001; thrombocytopenia: OR 0.345, p < 0.001) or platinum/vinorelbine (i.e., neutropenia: OR 0.360, p = 0.046; anemia: OR 0.181, p = 0.014) in matched patients. Further conventional logistic regression analyses indicated that pemetrexed/platinum was ranked lowest for the risks of early treatment discontinuation (OR 0.326, p < 0.001), treatment failure (OR 0.460, p < 0.001), and any hematological AE (OR 0.329, p < 0.001).

**Conclusions:**

Pemetrexed plus platinum had significantly superior clinical effectiveness as compared to the other platinum-based doublets with third-generation cytotoxic agents and was also associated with several lower hematological toxicity rates than gemcitabine or vinorelbine-based doublet in the first-line setting for advNS-NSCLC in Chinese patients.

## Background

The incidence of lung cancer in China has doubled in the past decade [1] likely in part due to the aging population, poorly controlled cigarette smoking, and worsening air pollution associated with industrialization [2, 3]. Non-small cell lung cancer (NSCLC) accounts for over 80% of lung cancer cases in China [4]. Because of the challenges associated with tumor early detection [5], more than half of Chinese patients with lung cancer are diagnosed at an advanced stage [6], which is not curable and is associated with a 5-year survival rate of less than 10% [7]. Thus, chemotherapy is the main therapeutic option to extend survival and to improve the quality of life in Chinese patients with advanced lung cancer [8].

The role of tumor histology in predicting treatment response to chemotherapy for advanced NSCLC was first suggested by a retrospective analysis reporting that pemetrexed was associated with superior effects than docetaxel in the second-line setting for advanced non-squamous NSCLC (advNS-NSCLC) [9]. Another retrospective analysis based on a phase III trial comparing cisplatin/pemetrexed versus cisplatin/gemcitabine in the first-line setting for advanced NSCLC also reported significantly better treatment response associated with pemetrexed treatment [10]. However, the superior treatment response associated with pemetrexed treatment for advNS-NSCLC has neither been confirmed in real-world study settings nor in patients of non-Caucasian race. In addition, to our knowledge, pemetrexed has never been compared with other third-generation cytotoxic agents for clinical effectiveness and clinical toxicity in the first-line setting for advNS-NSCLC in Chinese patients. Thus, we conducted this retrospective cohort study to assess clinical effectiveness and clinical toxicity associated with platinum-based doublets in the first-line setting for advNS-NSCLC to confirm previously reported data demonstrating significantly improved tumor response associated with pemetrexed treatment in the second-line setting [11] and to provide general additional clinic evidence to support treatment decision making in the first-line setting for advNS-NSCLC.

## Methods

This retrospective cohort study selected two tertiary care hospitals [Chinese Academy of Medical Sciences Tumor Hospital (CAMSTH) and Xuanwu Hospital (XWH)] for cancer care in Beijing (the national capital city of China, 2012 gross domestic product (GDP) per capita US$ 13,797) [12] and two tertiary care hospitals [Hunan Province Tumor Hospital (HNPTH), and Xiangya Hospital (XYH)] for cancer care in Changsha (the capital city of Hunan province, an inland province in southeastern China, 2012 GDP per capita US$ 5,304) (12) for case identification in order to create a study cohort reflecting the overall current social economic and referral patterns of lung cancer patients in China. This study was approved by the ethics committees of CAMSTH, XWH, HNPTH, and XYH, respectively.

### Patient identification

Data were obtained from electronic hospital admission registry databases in the selected two tertiary care hospitals located in Beijing (CAMSTH and XWH) and two tertiary care hospitals located in Changsha (HNPTH and XYH). The period for identifying eligible cases was set from January 1, 2007 to December 31, 2012. However, the searching time periods for CAMSTH and XYH started from January 1, 2009 and January 1, 2010, respectively, because the electronic hospital admission registry database did not contain data prior to these dates. Eligible cases were required to have a confirmed diagnosis of non-squamous non-small cell lung cancer, be diagnosed with Stage IIIB-IV disease, and have been treated with first-line platinum-based doublet therapy including pemetrexed (approved for second-line therapy in 2005 and subsequently approved for first-line chemotherapy in 2008), docetaxel, paclitaxel, gemcitabine, or vinorelbine. The platinum agent was limited to cisplatin or carboplatin, the two most frequently used platinum agents in the first-line setting for advanced NSCLC in China [8]. To identify eligible cases, the diagnostic fields of hospital admission registry databases were searched using keywords including “lung cancer”, “NSCLC”, “small cell lung cancer”, “non-squamous NSCLC”, “adenocarcinoma”, “large-cell lung cancer”, and “squamous NSCLC”. After exclusion of patients with a diagnosis of small cell lung cancer or squamous lung cancer, the identified patients with non-squamous NSCLC or histologically unclassified lung cancer were linked with their latest hospital records to confirm their tumor histology. Hospital records of the patients with biopsy or cytology-confirmed non-squamous NSCLC were further reviewed to exclude patients with tumor stage less than stage IIIB and patients who did not receive first-line chemotherapy for their advanced lung cancer. Finally, patients who received any tyrosine kinase inhibitor (TKI), epidermal growth factor receptor (EGFR) monoclonal antibody, or anti-angiogenic therapy in the first-line setting were excluded to control their confounding effects on outcome measures.

### Data extraction

The follow-up time for data extraction in our study was set from the admission date of the hospitalization initializing first-line chemotherapy to the discharge date of hospitalization with the last administration of first-line chemotherapy. The medical records associated with the first hospitalization were reviewed to extract each eligible patient’s baseline characteristics including demographics (gender, age), medical insurance type, smoking status prior to lung cancer diagnosis, Eastern Cooperative Oncology Group (ECOG) performance status, baseline laboratory blood testing for hemoglobin level, white blood cell (WBC) count, neutrophil granulocytes count, and platelet count prior to administration of chemotherapy regiments, tumor stage, tumor histology, number of metastatic sites, and metastasis locations. The prescription records associated with identified hospitalizations were reviewed to extract doses and administration schedules of chemotherapeutic agents. Medical records associated with hospitalizations during follow-up and after the completion of first-line chemotherapy were reviewed to extract tumor response information from each assessment related to first-line chemotherapy. The tumor response assessment in the four participating hospitals was based on the Response Evaluation Criteria in Solid Tumors (RECIST) evaluation criteria [13]. Chemotherapy adverse events (AE) report forms associated with each hospitalization during follow-up were reviewed to extract the information on the occurrences and severity of AEs. Common Terminology Criteria for Adverse Events (CTCAE) 3.0 with modifications on anemia for Chinese patients [14] was used to grade AEs in the four participating hospitals. Hospital records for laboratory blood testing during follow-up were reviewed to extract reported hemoglobin level, WBC count, neutrophilic granulocyte count, and platelet count as additional information to assess occurrences and severity of hematological AEs. Hospital medication prescription records during follow-up were also reviewed to extract information on the usages of medications (granulocyte colony-stimulating factor, G-CSF; erythropoietin, EPO; interleukin 11, IL-11; and thrombopoietin, TPO) and blood products (red blood cell and platelet) used for preventing and treating hematological AEs. Finally, hospital admission and discharge dates associated with each hospitalization during follow-up were collected to calculate the length of hospital stay.

### Outcome measures

Tumor response and the occurrences of AEs associated with studied platinum-based doublets were the primary outcome measures in this study. Because complete first-line chemotherapy usually requires 4 to 6 treatment cycles, early treatment discontinuation was defined as completed treatment cycles less than 4 in our study. The latest tumor response assessment based on RECIST after the completion of chemotherapy was used to determine the treatment response associated with the five studied platinum-based doublets. Our study also defined disease control [complete response (CR), partial response (PR), or stable disease (SD)] and treatment failure [progressive disease (PD) or early treatment discontinuation] for the comparisons of clinical effectiveness among the studied doublets. The identified AEs associated with the studied platinum-based doublets during follow-up were classified as hematological and non-hematological. In order to reduce the risk of missing information on AE assessment, the recorded hematological AEs in medical notes and the hematological AEs identified from blood laboratory testing records during follow-up were used to determine occurrence and severity of hematological AE associated with studied platinum-based doublets. The assessment of non-hematological AEs was only based on recorded AEs in medical records associated with included hospitalizations. The measured secondary outcomes in our study included the number of completed treatment cycles and average length of hospital stay per treatment cycle.

### Data analysis

Descriptive statistical methods were used to summarize patient baseline characteristics, selected platinum agent in doublet, and hematological AE management associated with five studied platinum-based doublets. One-way ANOVA analyses and chi square tests were used to examine the differences in patient baseline characteristics across the five treatment groups. Propensity score methods and conventional regression methods were used respectively to assess early treatment discontinuation, tumor response, disease control, treatment failure, and clinical toxicity associated with the studied platinum-based doublets. Propensity score methods created best matched pairs on patient baseline characteristics, platinum agent used in doublet, and hematological AE management for platinum plus pemetrexed versus the other four platinum-based doublets, respectively, using greedy approach [15] with matching condition of propensity score difference between matched pair less than 0.001. Paired t-test and McNemar’s test were used to compare the matched treatment groups to assess the balance of patient baseline characteristics and the differences in number of completed treatment cycles, average length of hospital stay per treatment cycle, tumor response based on RECIST evaluation criteria, early treatment discontinuation, disease control, treatment failure, and AEs associated with chemotherapy. Further multiple logistic regression analyses with generalized estimating equation adjusted imbalanced baseline variables (p < 0.5 after matching) in propensity score matched patients [16] to confirm the head-to-head comparisons of early treatment discontinuation, treatment failure, and hematological AEs. Finally, our study used conventional multiple logistic regression analyses to rank the impact of the five studied doublets using vinorelbine/platinum as reference on the risks of early treatment discontinuation, treatment failure, and hematological AEs after the adjustment of patient baseline characteristics, platinum agent used in doublet, and hematological AE management. Statistical significance in our study was defined as two-sided p value less than 0.05 and SAS 9.2 was used to perform the data analyses as described above.

## Results

**Figure 1:**
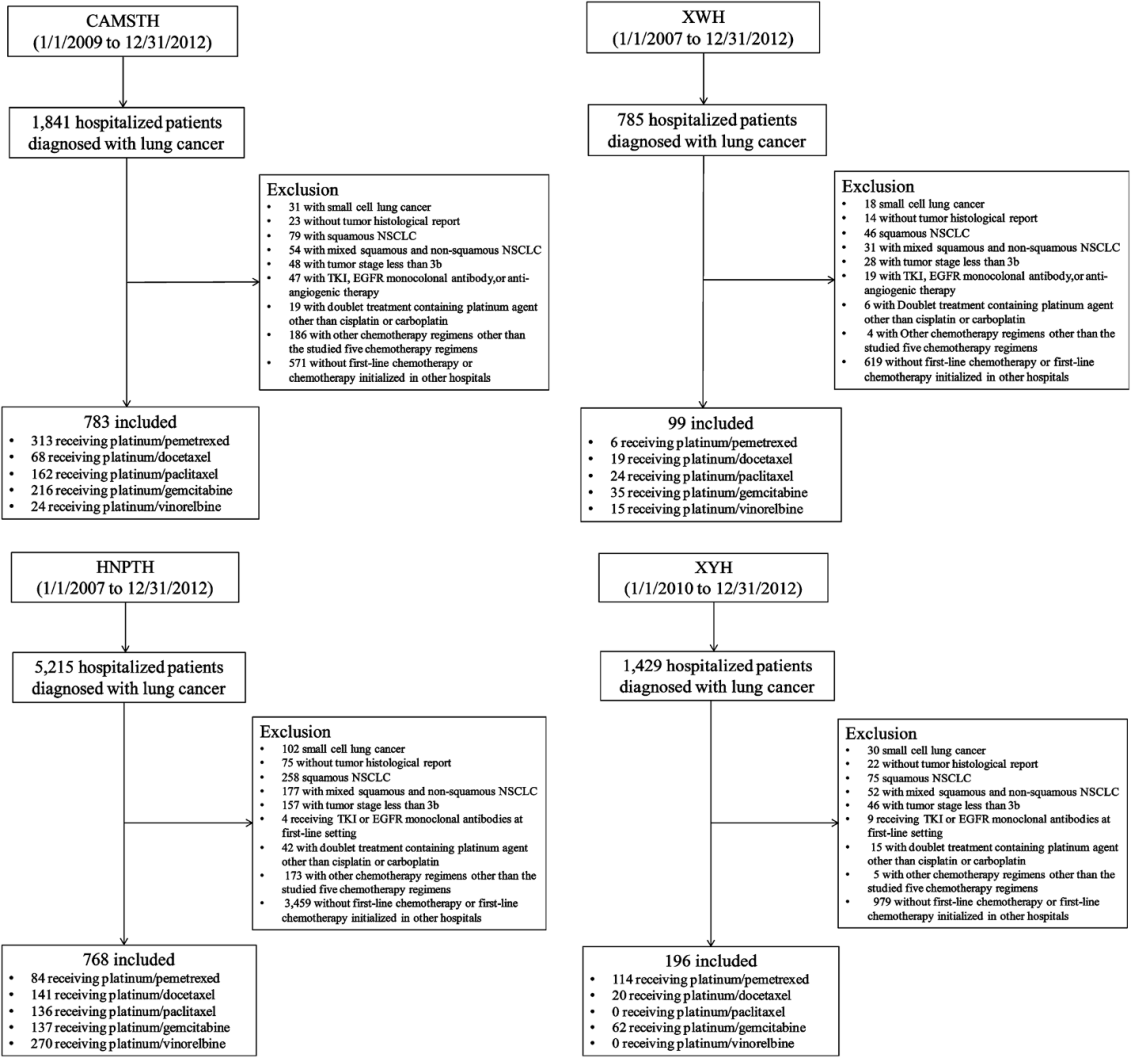
**Flow chart to identify eligible patients in the four participating tertiary care hospitals.** *Abbreviations:* CAMSTH, Chinese Academy of Medical Sciences Tumor Hospital; XWH, Xuanwu Hospital; HNPTH, Hunan Province Tumor Hospital; XYH, Xiangya Hospital; TKI, tyrosine kinase inhibitor; EGFR, epidermal growth factor receptor.

The initial screening hospital admission registry databases of the four selected hospitals identified 9,270 patients with lung cancer. After excluding134 patients without tumor histology information, 279 patients with tumor stage less than IIIB, 181 patients with small cell lung cancer, 458 patients with squamous NSCLC, 314 patients with mixed squamous and non-squamous NSCLC, 5,529 patients who did not receive first-line treatment or started first-line treatment in other hospitals, 368 patients receiving first-line chemotherapy regimens other than the five studied platinum-based doublets, 79 patients receiving TKI, EGFR monoclonal antibody, or anti-angiogenic therapy in the first-line setting, and 82 patients receiving platinum-based doublets containing platinum agent that was not cisplatin or carboplatin, there were a total of 1,846 eligible patients included in the data analysis. The patient identification flow charts in the four hospitals are illustrated in Figure 1.

### Patient baseline characteristics, platinum agent in doublet, and hematological AE management

Of the included 1,846 patients, 517 patients received platinum/pemetrexed, 248 patients received platinum/docetaxel, 322 patients received platinum/paclitaxel, 450 patients received platinum/gemcitabine, and 309 patients received platinum/vinorelbine. Many patient baseline characteristics did not vary substantially across the five platinum-based doublets (Table 1). The identified baseline characteristics with significant differences across the five treatment groups included age (mean from 53.5 to 55.5 years, p = 0.019), body surface area (mean from 1.6 to 1.7 m^2^, p < 0.001), non-smoking prevalence rates (from 43.2% to 56.3%, p = 0.003), distribution of public health insurance plan for urban (from 42.7% to 59.4%, p < 0.001) and rural residents (from 17.2% to 40.8%, p < 0.001), distribution of ECOG performance status of 0 (from 19.4% to 39.8%, p < 0.001) and 1 (from 58.8% to 76.7%, p < 0.001), baseline hemoglobin (mean from 129.4 to 132.9 g/l, p = 0.043), and distributions of tumor stage IV (from 80.4% to 92.5%, p < 0.001) and pleural metastasis (from 11.3% to 24.8%, p < 0.001).

**Table 1:** **Patient baseline characteristics, selection of the platinum agent, and hematological AE management of studied doublets**

Platinum-based doublet	Platinum/Pemetrexed			Platinum/Docetaxel			Platinum/Paclitaxel			Platinum/Gemcitabine			Platinum/Vinorelbine			
Sample size	517			248			322			450			309			P value*
Variables	N	Mean/%	STDEV	N	Mean/%	STDEV	N	Mean/%	STDEV	N	Mean/%	STDEV	N	Mean/%	STDEV	
*Demography*																
Age (years)	511	55.5	9.8	248	54.1	9.9	322	53.8	10.5	447	55.1	9.4	308	53.5	10.2	***0.019***
BMI	441	23.6	3.2	136	23.9	3.2	214	23.5	3.3	332	23.7	3.3	103	23.0	3.4	0.228
BSA (m^2^)	457	1.7	0.2	214	1.7	0.2	276	1.7	0.2	395	1.7	0.2	266	1.6	0.2	***<0.001***
Male (%)	303	58.6%	—	166	66.9%	—	194	60.3%	—	296	65.8%	—	187	60.5%	—	0.074
Non-smoking (%)	291	56.3%	—	107	43.2%	—	173	53.7%	—	211	46.9%	—	159	51.5%	—	***0.003***
*Public health insurance plan (%)*																
Urban residents	307	59.4%	—	132	53.2%	—	166	51.6%	—	231	51.3%	—	132	42.7%	—	***<0.001***
Rural residents	89	17.2%	—	65	26.2%	—	76	23.6%	—	108	24.0%	—	126	40.8%	—	***<0.001***
ECOG performance status (%)																
0	193	37.3%	—	58	23.4%	—	128	39.8%	—	133	29.6%	—	60	19.4%	—	***<0.001***
1	304	58.8%	—	180	72.6%	—	190	59.0%	—	299	66.4%	—	237	76.7%	—	***<0.001***
2	13	2.5%	—	9	3.6%	—	3	0.9%	—	13	2.9%	—	10	3.2%	—	0.265
*Baseline marrow function*																
Hemoglobin (g/l)	495	132.9	17.6	241	131.0	16.5	317	132.8	15.3	426	132.5	19.3	304	129.4	17.3	***0.043***
Neutrophilic granulocyte count ( ×10^9^/l)	491	5.2	2.4	239	4.9	2.2	318	5.2	2.3	422	5.2	2.6	304	4.9	2.0	0.112
WBC ( ×10^9^/l)	495	7.6	2.6	242	7.4	2.8	318	7.6	2.7	428	7.8	2.8	305	7.5	2.4	0.601
Platelet count ( ×10^10^/l)	493	24.9	8.0	241	25.6	9.4	317	25.9	8.6	424	25.0	25.8	304	24.8	8.2	0.213
*Tumor stage and histology (%)*																
Stage 4	478	92.5%	—	215	86.7%	—	273	84.8%	—	395	87.8%	—	248	80.4%	—	***<0.001***
Adenocarcinoma type	512	99.0%	—	241	97.2%	—	313	97.2%	—	440	97.8%	—	307	99.3%	—	0.272
*Number of metastasis site (%)*																
1	250	48.4%	—	107	43.2%	—	143	44.4%	—	100	22.2%	—	153	49.7%	—	0.513
2	123	23.8%	—	49	19.8%	—	70	21.7%	—	211	46.9%	—	66	21.2%	—	0.758
3 or above	64	12.4%	—	20	8.1%	—	32	9.9%	—	100	22.2%	—	22	7.2%	—	0.083
*Location of metastasis (%)*																
Brain	84	16.3%	—	49	19.8%	—	58	18.0%	—	68	15.1%	—	42	13.7%	—	0.289
Bone	200	38.7%	—	87	35.1%	—	98	30.4%	—	162	36.0%	—	109	35.3%	—	0.199
Liver	48	9.3%	—	23	9.3%	—	29	9.0%	—	32	7.1%	—	24	7.8%	—	0.729
Pleural	128	24.8%	—	28	11.3%	—	50	15.5%	—	80	17.8%	—	45	14.7%	—	***<0.001***
*Platinum agent used in doublet*																
Cisplatin	400	77.4%	—	111	44.7%	—	251	77.8%	—	385	85.6%	—	272	87.9%	—	***<0.001***
Carboplatin	117	22.6%	—	137	55.3%	—	71	22.2%	—	65	14.4%	—	37	12.1%	—	***<0.001***
*Hematological AE management*																
G-CSF	172	33.3%	—	108	43.5%	—	167	51.9%	—	209	46.4%	—	215	69.6%	—	***<0.001***
EPO	5	1.0%	—	1	0.3%	—	0	0.0%	—	9	2.0%	—	3	1.0%	—	0.066
IL-11	16	3.1%	—	5	1.9%	—	3	0.9%	—	44	9.8%	—	39	12.6%	—	***<0.001***
TPO	17	3.3%	—	2	0.6%	—	0	0.0%	—	9	2.0%	—	3	1.0%	—	***0.003***
RBC	1	0.2%	—	0	0.0%	—	0	0.0%	—	5	1.1%	—	8	2.6%	—	***<0.001***
Platelet	1	0.2%	—	1	0.3%	—	0	0.0%	—	3	0.7%	—	4	1.3%	—	0.148

AE, adverse event; STDEV, standard deviation; BMI, body mass index; BSA, body surface area; ECOG, Eastern Cooperative Oncology Group; WBC, white blood cell; G-CSF, granulocyte colony-stimulating factor; EPO, erythropoietin; IL-11, interleukin 11; TPO, thrombopoietin; RBC, red blood cell. *: P values less than 0.05 were in bold to indicate significant differences.

Comparisons of the distribution of selected platinum agent contained in the studied doublets indicated that cisplatin was used most often in patients receiving platinum/vinorelbine (87.9%, p < 0.001) and carboplatin was used most often in patients treated by platinum/docetaxel (55.3%, p < 0.001). Further comparisons of the distributions of medications and blood products used for treating hematological AEs suggested that G-CSF was the most frequently used medication (ranged from 33.3% in patients receiving platinum/pemetrexed to 69.6% in patients receiving platinum/vinorelbine, p < 0.001). The uses of EPO (n = 18, 1.0%), IL-11 (n = 107, 5.8%), and TPO (n = 31, 1.7%) in our study cohort were much less prevalent than the use of G-CSF. Blood transfusion (n = 14, 0.9%) and platelet infusion (n = 9, 0.5%) was rarely used in our study cohort. The distributions of selected platinum agent in the studied doublets and hematological AE-related treatments are also summarized in Table 1.

### Head-to-head comparisons of clinical effectiveness and clinical toxicity between the propensity score matched treatment groups

Propensity score methods created 95 best matched pairs for platinum/pemetrexed versus platinum/docetaxel, 118 best matched pairs for platinum/pemetrexed versus platinum/paclitaxel, 199 best matched pairs for platinum/pemetrexed versus platinum/gemcitabine, and 72 best matched pairs for platinum/pemetrexed versus platinum/vinorelbine for head-to-head comparisons of tumor response and clinical toxicity.

#### Clinical effectiveness

The distribution of cisplatin and carboplatin were well balanced in the created matched treatment groups for all comparisons. The five studied platinum-based doublets in the matched patients were administrated every three weeks and the average dosages of the five cytotoxic agents per treatment cycle in the matched patients were similar to what were recommended in clinical guidelines. When compared to other four studied doublets, platinum/pemetrexed was associated with significantly more completed treatment cycles (mean differences: 0.5 cycles for the comparison with platinum/docetaxel, p = 0.028 to 0.9 cycles for the comparison with platinum/gemcitabine, p < 0.001) and significantly lower early treatment discontinuation rate (rate ratio (RR): 0.674 for the comparison with platinum/gemcitabine, p < 0.001 to 0.732 for the comparison with platinum/paclitaxel, p = 0.022). Additionally, platinum/pemetrexed was associated with significantly shorter length of hospital stay per treatment cycle than paclitaxel (10.4+/-6.1 days vs. 13.5+/-11.7 days, p = 0.011), gemcitabine (10.9+/-6.0 days vs. 15.0+/-7.0 days, p < 0.001), or vinorelbine-contained doublet (13.1+/-7.2 days vs. 16.9+/-8.5 days, p = 0.001).

**Figure 2:**
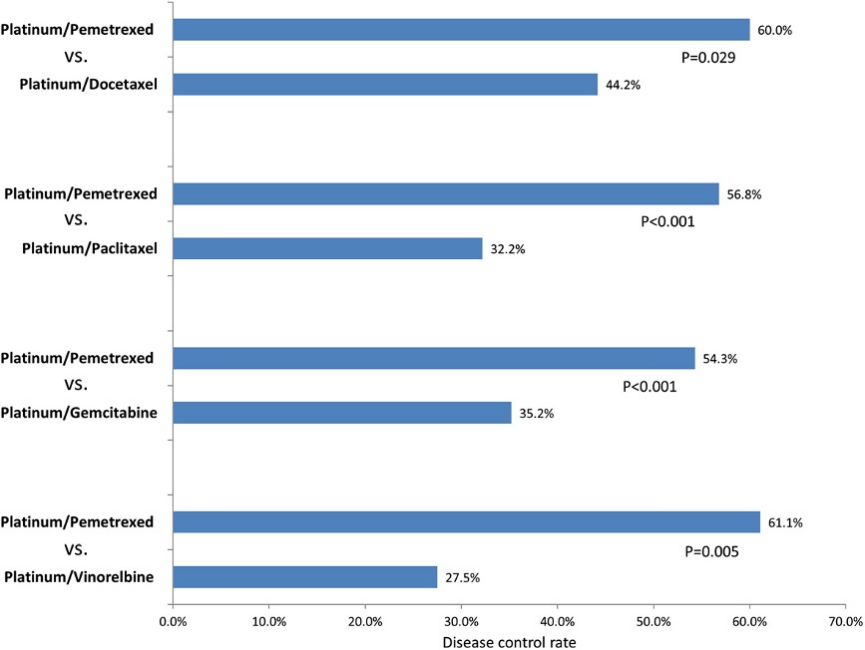
**Comparisons of disease control rate (partial response or stable disease reported by tumor response assessment) between platinum/pemetrexed and the other four studied platinum-based doublets in the propensity score matched patients.**

Our study compared the distribution of tumor response assessed by RECIST between platinum/pemetrexed and the other four studied doublets after the completions of treatment cycles (3.33 to 3.58 cycles for platinum/pemetrexed, 2.89 cycles for platinum/docetaxel, 2.93 cycles for platinum/paclitaxel, 2.71 cycles for platinum/gemcitabine, 2.49 cycles for platinum/vinorelbine) in propensity score matched patients. Our study didn’t identify any CR associated with the five studied platinum-based doublets in the propensity score matched patients. Comparisons of the distributions of tumor response classified by RECIST observed significantly higher rates of PR associated with platinum/pemetrexed when compared with paclitaxel (18.6% vs. 5.1%, RR 3.647, p = 0.002) or gemcitabine-contained doublet (18.1% vs. 9.1%, RR 1.989, p = 0.007). Platinum/pemetrexed was also associated with significantly more patients with SD than docetaxel (46.3% vs. 30.5%, RR 1.518, p = 0.025) or gemcitabine-contained doublet (36.2% vs. 26.1%, RR 1.387, p = 0.043). Thus, platinum/pemetrexed was associated with significantly higher disease control rate (RR: 1.357, p = 0.029 for the comparison with platinum/docetaxel to 2.222, p = 0.005 for the comparison with platinum/vinorelbine) than the other four studied doublets in propensity score matched patients (Figure 2). However, no significant differences were identified for the comparisons of PD between platinum/pemetrexed and the other four studied doublets likely because of a large proportion of patients who did not have tumor response assessment information due to early treatment discontinuation. When using treatment failure as the outcome to assess clinical effectiveness, platinum/pemetrexed was associated with significantly lower rate of treatment failure (RR: 0.537, p = 0.005 for the comparison with platinum/vinorelbine to 0.717, p = 0.029 for the comparison with platinum/docetaxel) than the other four studied doublets. The results of head-to-head comparisons of treatment pattern and clinical effectiveness between propensity score matched treatment groups are summarized in Table 2. With further adjustment of imbalanced baseline variables after propensity score matching, platinum/pemetrexed was confirmed to have significantly lower risks of early treatment discontinuation (odds ratio (OR): 0.239, p = 0.001 for the comparison with platinum/docetaxel to 0.389, p = 0.003 for the comparison with platinum/paclitaxel) and treatment failure (OR: 0.257, p < 0.001 for the comparison with platinum/paclitaxel to 0.381, p < 0.001 for the comparison with platinum/gemcitabine) than the other four studied doublets (Table 3).

**Table 2:** **Head-to-head comparisons of treatment pattern and tumor response between the propensity score matched treatment groups**

Comparison	Platinum/Pemetrexed vs. Platinum/Docetaxel								Platinum/Pemetrexed vs. Platinum/Paclitaxel								Platinum/Pemetrexed vs. Platinum/Gemcitabine								Platinum/Pemetrexed vs. Platinum/Vinorelbine							
Matched pairs	95						RR	P value*	118						RR	P value*	199						RR	P value*	72						RR	P value*
Measured outcomes	N	Mean/%	STDEV	N	Mean/%	STDEV			N	Mean/%	STDEV	N	Mean/%	STDEV			N	Mean/%	STDEV	N	Mean/%	STDEV			N	Mean/%	STDEV	N	Mean/%	STDEV		
*Treatment pattern*																																
Completed treatment cycles	95	3.4	1.7		95	2.9	1.7	***0.028***	118	3.6	1.7	118	2.9	1.6		***0.006***	199	3.6	1.7	199	2.7	1.5		***<0.001***	72	3.3	1.7	72	2.5	1.3		***0.003***
Length of hospital stay per cycle (days)	95	10.1	6.7	95	11.5	8.2		0.191	118	10.4	6.1	118	13.5	11.7		***0.011***	199	10.9	6.0	199	15.0	7.0		***<0.001***	72	13.1	7.2	72	16.9	8.5		***0.001***
*Tumor response based on RECIST (%)*																																
PR	13	13.7%		13	13.7%		1.000	1.000	22	18.6%		6	5.1%		3.647	***0.002***	36	18.1%		18	9.1%		1.989	***0.007***	16	22.2%		9	12.5%		1.776	0.108
SD	44	46.3%		29	30.5%		1.518	***0.025***	45	38.1%		32	27.1%		1.406	0.074	72	36.2%		52	26.1%		1.387	***0.043***	28	38.9%		18	25.0%		1.556	0.077
PD	9	9.5%		13	13.7%		0.693	0.371	11	9.3%		21	17.8%		0.522	0.059	24	12.1%		23	11.6%		1.043	0.879	7	9.7%		12	16.7%		0.581	0.197
Unknown	29	30.5%		40	42.1%		0.724	0.101	40	33.9%		59	50.0%		0.678	***0.020***	67	33.7%		106	53.3%		0.632	***<0.001***	21	29.2%		33	45.8%		0.638	***0.029***
*Other tumor response outcomes (%)*																																
Early treatment discontinuation	45	47.4%		62	65.3%		0.726	***0.007***	52	44.1%		71	60.2%		0.732	***0.022***	91	45.7%		135	67.8%		0.674	***<0.001***	38	52.8%		55	76.4%		0.691	***0.007***
Tumor control	57	60.0%		42	44.2%		1.357	***0.029***	67	56.8%		38	32.2%		1.764	***<0.001***	108	54.3%		70	35.2%		1.543	***<0.001***	44	61.1%		20	27.5%		2.222	***0.005***
Treatment failure	38	40.0%		53	55.8%		0.717	***0.029***	51	43.2%		80	67.8%		0.637	***<0.001***	91	45.7%		129	64.8%		0.705	***<0.001***	28	38.9%		45	72.5%	0.537		***0.005***

STDEV, standard deviation; RR, rate ratio; RECIST, Response Evaluation Criteria in Solid Tumors; PR, partial response; SD, stable disease; PD, progressive disease. *: P values less than 0.05 were in bold to indicate significant differences.

**Table 3:** **Comparing early treatment discontinuation, treatment failure, and hematological AEs between matched treatment groups after adjusting imbalanced baseline variables**

Treatment comparison	Platinum/Pemetrexed vs. Platinum/Docetaxel				Platinum/Pemetrexed vs. Platinum/Paclitaxel				Platinum/Pemetrexed vs. Platinum/Gemcitabine				Platinum/Pemetrexed vs. Platinum/Vinorelbine			
Sample size	190				236				398				144			
Outcome measures	OR	95% CI		P value*	OR	95% CI		P value*	OR	95% CI		P value*	OR	95% CI		P value*
		Lower limit	Upper limit			Lower limit	Upper limit			Lower limit	Upper limit			Lower limit	Upper limit	
Early treatment discontinuation	0.239	0.106	0.536	***0.001***	0.389	0.210	0.722	***0.003***	0.340	0.215	0.538	***<0.001***	0.250	0.096	0.654	***0.005***
Treatment failure	0.377	0.189	0.750	***0.006***	0.257	0.134	0.493	***<0.001***	0.381	0.241	0.601	***<0.001***	0.265	0.109	0.647	***0.004***
*Hematological AE*																
Neutropenia	2.140	0.738	6.205	0.161	0.695	0.352	1.372	0.295	1.300	0.789	2.143	0.304	0.360	0.132	0.981	***0.046***
Leukopenia	2.028	0.691	5.948	0.198	0.687	0.351	1.345	0.273	0.633	0.392	1.023	0.062	0.423	0.165	1.082	0.073
Anemia	0.905	0.404	2.026	0.808	0.618	0.326	1.174	0.142	0.357	0.221	0.576	***<0.001***	0.181	0.046	0.710	***0.014***
Thrombocytopenia	0.790	0.287	2.178	0.649	0.581	0.287	1.174	0.130	0.345	0.209	0.570	***<0.001***	1.105	0.413	2.960	0.842
Any hematological AE	0.687	0.327	1.442	0.321	0.508	0.274	0.943	***0.032***	0.383	0.229	0.642	***<0.001***	0.332	0.092	1.200	0.093

AE, adverse event; OR, odds ratio; CI, confidence interval. *: P values less than 0.05 were in bold to indicate significant differences.

#### Clinical toxicity

The distribution of hematological AEs associated with platinum/pemetrexed was not significantly different from platinum/docetaxel or platinum/paclitaxel. However, platinum/pemetrexed was associated with significantly lower rates of leukopenia (32.2% vs. 42.2%, RR 0.762, p = 0.041), anemia (29.6% vs. 50.3%, RR 0.590, p < 0.001), and thrombocytopenia (43.7% vs. 62.8%, RR 0.696, p < 0.001) than platinum/gemcitabine and significantly lower rate of neutropenia (16.7% vs. 31.9%, RR 0.522, p = 0.034) than platinum/vinorelbine (Table 4). With further adjusting imbalanced baseline variables after propensity score matching, platinum/pemetrexed was confirmed to have a comparable hematological toxicity profile as platinum/docetaxel but significantly less hematological toxicity than paclitaxel (any hematological AE: OR 0.508, p = 0.032), gemcitabine (anemia: OR 0.357, p < 0.001; thrombocytopenia: OR 0.345, p < 0.001; any hematological AE: OR 0.383, p < 0.001), or vinorelbine-contained doublet (neutropenia: OR 0.360, p = 0.046; anemia: OR 0.181, p = 0.014) (Table 3).

**Table 4:** **Comparing occurrences of hematological and non-hematological AEs between the propensity score matched treatment groups**

Treatment comparison	Platinum/Pemetrexed vs. Platinum/Docetaxel						Platinum/Pemetrexed vs. Platinum/Paclitaxel						Platinum/Pemetrexed vs. Platinum/Gemcitabine						Platinum/Pemetrexed vs. Platinum/Vinorelbine					
Matched pairs	95				RR	P value*	118				RR	P value*	199				RR	P value*	72				RR	P value*
All grades AE	N	%	N	%			N	%	N	%			N	%	N	%			N	%	N	%		
*Hematological AE*																								
Leukopenia	29	30.5%	27	28.4%	1.074	0.752	34	28.8%	39	33.1%	0.872	0.508	64	32.2%	84	42.2%	0.762	***0.041***	18	25.0%	27	37.5%	0.667	0.106
Neutropenia	24	25.3%	19	20.0%	1.263	0.336	29	24.6%	35	29.7%	0.829	0.396	63	31.7%	57	28.6%	1.105	0.540	12	16.7%	23	31.9%	0.522	***0.034***
Thrombocytopenia	24	25.3%	24	25.3%	1.000	1.000	32	27.1%	34	28.8%	0.941	0.773	59	29.6%	100	50.3%	0.590	***<0.001***	25	34.7%	26	36.1%	0.962	0.876
Anemia	34	35.8%	31	32.6%	1.097	0.647	46	39.0%	53	44.9%	0.868	0.370	87	43.7%	125	62.8%	0.696	***<0.001***	35	48.6%	46	63.9%	0.761	0.063
Any hematological AE	56	58.9%	58	61.1%	0.966	0.752	70	59.3%	80	67.8%	0.875	0.189	130	65.3%	163	81.9%	0.798	***<0.001***	47	65.3%	58	80.6%	0.810	0.056
*Non-hematological AE*																				*0.0%*		*0.0%*		
Alopecia	3	3.2%	15	15.8%	0.200	***0.005***	1	0.8%	8	6.8%	0.125	***0.020***	2	1.0%	18	9.0%	0.111	***<0.001***	0	0.0%	1	1.4%	0.000	1.000
Arthralgia	5	5.3%	4	4.2%	1.250	0.706	0	0.0%	0	0.0%	—	—	2	1.0%	8	4.0%	0.250	0.058	0	0.0%	0	0.0%	—	—
Cough	1	1.1%	2	2.1%	0.500	0.564	1	0.8%	1	0.8%	1.000	1.000	3	1.5%	12	6.0%	0.250	***0.020***	2	2.8%	1	1.4%	2.000	0.564
Dermatitis	0	0.0%	0	0.0%	—	—	0	0.0%	0	0.0%	—	—	0	0.0%	0	0.0%	—	—	0	0.0%	0	0.0%	—	—
Diarrhea	1	1.1%	2	2.1%	0.500	0.317	1	0.8%	0	0.0%	—	1.000	3	1.5%	0	0.0%	—	0.250	2	2.8%	3	4.2%	0.667	0.655
Dyspnea	1	1.1%	0	0.0%	—	1.000	1	0.8%	0	0.0%	—	1.000	3	1.5%	6	3.0%	0.500	0.317	1	1.4%	0	0.0%	—	1.000
Edema	1	1.1%	0	0.0%	—	1.000	1	0.8%	0	0.0%	—	1.000	3	1.5%	1	0.5%	3.000	0.317	1	1.4%	0	0.0%	—	1.000
Fatigue	11	11.6%	11	11.6%	1.000	1.000	10	8.5%	27	22.9%	0.370	***0.001***	18	9.0%	62	31.2%	0.290	***<0.001***	13	18.1%	23	31.9%	0.565	0.048
Nausea	63	66.3%	67	70.5%	0.940	0.527	78	66.1%	84	71.2%	0.929	0.446	134	67.3%	148	74.4%	0.905	0.122	53	73.6%	53	73.6%	1.000	1.000
Peripheral neuropathy	1	1.1%	3	3.2%	0.333	0.317	0	0.0%	5	4.2%	0.000	0.063	3	1.5%	2	1.0%	1.500	0.657	1	1.4%	2	2.8%	0.500	0.564
Rash	2	2.1%	0	0.0%	—	0.500	2	1.7%	0	0.0%	—	0.500	5	2.5%	5	2.5%	1.000	1.000	1	1.4%	0	0.0%	—	1.000
Stomatitis	0	0.0%	0	0.0%	—	—	0	0.0%	0	0.0%	—	—	0	0.0%	1	0.5%	0.000	1.000	0	0.0%	2	2.8%	0.000	0.500
Vomiting	40	42.1%	47	49.5%	0.851	0.286	51	43.2%	52	44.1%	0.981	0.895	92	46.2%	94	47.2%	0.979	0.849	31	43.1%	37	51.4%	0.838	0.366
Weight loss	0	0.0%	1	1.1%	0.000	1.000	0	0.0%	0	0.0%	—	—	0	0.0%	0	0.0%	—	—	0	0.0%	0	0.0%	—	—

AE, adverse event; RR, rate ratio. *P values less than 0.05 were in bold to indicate significant differences.

Head-to-head comparisons of non-hematological AEs between propensity score matched treatment groups further observed that platinum/pemetrexed was associated with significantly fewer patients experiencing alopecia than docetaxel (3.2% vs. 15.8%, RR 0.200, p = 0.005), paclitaxel (0.8% vs. 6.8%, RR 0.125, p = 0.020), or gemcitabine-contained doublet (1.0% vs. 9.0%, RR 0.111, p < 0.001). Platinum/pemetrexed was also associated with less fatigue than paclitaxel (8.5% vs. 22.9%, RR 0.370, p = 0.001), gemcitabine (9.0% vs. 31.2%, RR 0.290, p < 0.001), or vinorelbine-contained doublet (18.1% vs. 31.9%, RR 0.565, p = 0.048). Finally, platinum/pemetrexed was associated with significantly lower rate of cough (1.5% vs. 6.0%, RR 0.250, p = 0.020) when compared with platinum/gemcitabine. All other non-hematologic toxicities were not significantly different (Table 4).

### Multiple logistic regression analyses assessing the risks of early treatment discontinuation, treatment failure, and hematological AEs

**Figure 3:**
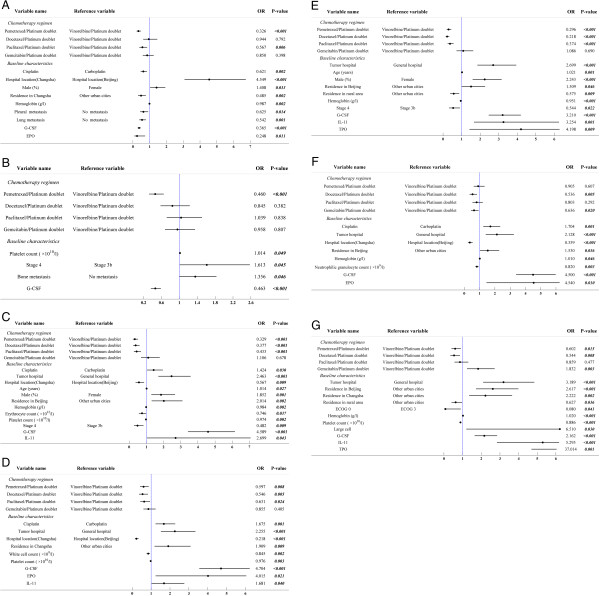
**Ranking the five studied doublets for tumor response and hematological AEs in 1691 included patients. a**. Early treatment discontinuation. **b**. Treatment failure. **c**. Any hematological AE. **d**. Leukopenia. **e**. Anemia. **f**. Neutropenia. **g**. Thrombocytopenia. *Abbreviations:* AE, adverse event; OR, odds ratio; G-CSF, granulocyte colony-stimulating factor; EPO, erythropoietin; IL-11, interleukin 11; ECOG, Eastern Cooperative Oncology Group; TPO, thrombopoietin. *Note:* Only baseline variables with significant OR and the studied doublets were displayed in graphs.

1,691 patients with complete information on patient baseline characteristics and hematological AE management were included for the assessment on the risks of early treatment discontinuation, treatment failure, and hematological AEs associated with the five studied platinum-based doublets. When platinum/vinorelbine was used as the reference, platinum/pemetrexed was associated with the lowest odds of early treatment discontinuation (OR 0.326, p < 0.001) (Figure 3a) and treatment failure (OR 0.460, p < 0.001) (Figure 3b). The only other doublet to differ significantly from platinum/vinorelbine regarding risk of early treatment discontinuation was the platinum/paclitaxel doublet (OR 0.567, p = 0.006). Docetaxel, paclitaxel, or gemcitabine-contained doublet did not differ significantly from platinum/vinorelbine regarding the odds of treatment failure. When compared to platinum/vinorelbine for the risks of hematological AEs, pemetrexed, docetaxel, and paclitaxel-contained doublets were associated with significantly reduced odds of experiencing any hematological AEs (OR ranged from 0.329 to 0.433, all p values <0.001) (Figure 3c), leukopenia (OR ranged from 0.546 to 0.631, p values ranged from 0.005 to 0.024) (Figure 3d), and anemia (OR ranged from 0.218 to 0.374, all p values < 0.001) (Figure 3e). Additionally, platinum/gemcitabine was associated with significantly reduced odds of neutropenia (OR 0.636, p = 0.020) (Figure 3f) but significantly greater thrombocytopenia (OR 1.832, p = 0.003) (Figure 3g) when compared with platinum/vinorelbine. Finally, pemetrexed (OR 0.602, p = 0.015) or docetaxel-contained doublet (OR 0.544, p = 0.008) had significantly lower odds of thrombocytopenia than platinum/vinorelbine (Figure 3g).

## Discussion

To our knowledge, our study is the first and the largest real-world study evaluating platinum-based doublets with pemetrexed and the other four third-generation doublets frequently used in the first-line setting for advNS-NSCLC. Our study used two statistical approaches, propensity score methods, and conventional regression methods, to compare clinical effectiveness and clinical toxicity associated with the studied five platinum-based doublets. Pemetrexed-based doublet was confirmed to be associated with significantly better tumor control than gemcitabine-based doublet when treating advNS-NSCLC in the first-line setting. Our study also generated evidence indicating that the pemetrexed treatment was associated with significantly lower odds of treatment failure versus other platinum doublets in the first-line setting for advNS-NSCLC. Further clinical toxicity assessment suggested that pemetrexed, docetaxel, and paclitaxel-contained doublets had comparable toxicity profiles, but gemcitabine and vinorelbine-contained doublets were significantly associated with greater toxicities than platinum/pemetrexed. Additionally, our study observed a significantly lower rate of early treatment discontinuation and shorter length of hospital stay per treatment cycle associated with pemetrexed treatment when compared with other doublets. It is possible that this could be due to differences in observed clinical effectiveness and clinical toxicity between the studied doublets.

Because pemetrexed in combination with cisplatin or carboplatin has been proven to yield efficacy results comparable with other platinum doublets in chemotherapy naïve patients with advanced NSCLC [17, 18], the observed superior clinical effectiveness associated with pemetrexed in our study may be explained by the potential impact of tumor histology on the anti-cancer mechanisms of the studied third-generation cytotoxic agents. Because docetaxel, paclitaxel, vinorelbine are mitotic inhibitors [19] and gemcitabine is a nucleoside analog [20], these four cytotoxic agents target common elements directly related to tumor cell replication and their anti-cancer activities are less sensitive to tumor histology. Pemetrexed inhibits three key enzymes in the folate metabolic pathway in tumor cells [21] and its anti-cancer activities are sensitive to expressions of enzymes in the folate metabolic pathway [22]. The observed superior clinical effectiveness associated with pemetrexed treatment in our study may be the result of highly prevalent adenocarcinoma histology (98.1%) in our study cohort, a histological subtype which expresses thymidylate synthase, one of the three key enzymes inhibited by pemetrexed [23]. In addition, the tumor response associated with platinum/pemetrexed in our study was increased by over half when compared with platinum/gemcitabine. However, the effects associated with pemetrexed treatment were only increased by about 10% for the same comparison in a Phase III trial mainly consisting Caucasian patients [10]. We also noticed that the treatment effectiveness associated with platinum/pemetrexed was much better than what was observed in PointBreak study [24], a phase III trial observing highly comparable disease control rates between carboplatin/pemetrexed and carboplatin/paclitaxel. We compared the patient baseline characteristics between the two studies and the differences in patient baseline characteristics may explain the differences in clinical findings in these two studies. First, the average age of patients in our study was about ten years younger than patients in the PointBreak study and younger age was likely to increase sensitivity of pemetrexed treatment [25]. Second, patients in our study mainly received cisplatin as platinum agent in the studied doublets and cisplatin-based chemotherapy has been proven to produce a higher response rate than carboplatin-based chemotherapy in a meta-analysis of eight trials including 2948 patients [26]. Third, almost all patients in our study cohort had adenocarcinoma lung cancer, which has lower expression of thymidylate synthase, a proven predictor for better response to pemetrexed treatment [27]. Finally, Chinese ethnicity in our study cohort could be another potential predictor to pemetrexed treatment as our previous study [11] also found much stronger response associated with pemetrexed treatment in the second-line setting in Chinese patients when compared to other studies mainly including Caucasian patients. Thus, the observed superior clinical effectiveness associated with pemetrexed treatment in our study should be further evaluated for their potential impact on overall survival in Chinese patients using a better study design and the possible predictive roles of age, platinum agent, thymidylate synthase, and Chinese ethnicity for the treatment response to pemetrexed in the first-line setting for advNS-NSCLC should be further investigated to guide future clinical practices.

Our study found that pemetrexed and docetaxel-contained doublets had comparable toxicity profiles. It is possible that the toxicity associated with a platinum agent could mask the differences in toxicity between pemetrexed and docetaxel. The significantly more completed treatment cycles associated with pemetrexed-based therapy may also have played a role in the toxicity comparison, as there is the chance of more occurrences of AEs with longer treatment duration; however, this analysis was not conducted in the current study. In our study, treatment with G-CSF was highly prevalent in patients receiving all the doublets; however, about half of patients used G-CSF with the paclitaxel and gemcitabine doublets, nearly 70% with vinorelbine, and approximately one-third with pemetrexed. Of note, neutropenia and leukopenia were identified in less than one-third of the paclitaxel, gemcitabine or vinorelbine patient cohorts. The rates of G-CSF use and rates of neutropenia and leukopenia associated with platinum/pemetrexed were comparable in our study; therefore we assumed that it was unlikely that prophylaxis treatment with G-CSF was applied to patients receiving pemetrexed treatment. Adjusting hematological AE management, mainly through adjusting the use of G-CSF, was crucial to control bias when assessing hematological toxicity and also treatment effectiveness in our study.

The evidence generated in our study has meaningful implications for clinical practice and future research. The superior clinical effectiveness associated with platinum/pemetrexed in our study could be used to further support the role of tumor histology in guiding individualized chemotherapy in the first-line setting for advanced NSCLC. Our study has also filled current evidence gap in managing advNS-NSCLC as there is a lack of real-world evidence comparing pemetrexed treatment with docetaxel, paclitaxel, or vinorelbine doublets. The strong treatment response associated with pemetrexed in our study further supported the hypothesis regarding the possible predictive role of Chinese ethnicity and future studies are needed to confirm this hypothesis. Different from chemotherapy care in high-income countries, chemotherapy care in China is usually conducted in hospital settings to monitor and manage chemotherapy toxicity. The reported shorter length of hospital stay associated with pemetrexed treatments is expected to reduce health resources utilization and have a positive impact on patient quality of life. Thus, any additional benefits associated with pemetrexed treatment on health resource utilization and quality of life should be further clarified as these two outcomes have been increasingly used to support both treatment and reimbursement decision makings. Finally, the observed highly prevalent use of G-CSF in our study suggests that prophylaxis treatment is common in real-world settings and adjusting the use of G-CSF is needed to minimize bias in assessing chemotherapy in real-world studies.

Our study has limitations commonly associated with retrospective studies. Therefore, these study results should be carefully interpreted. First, our study was unable to identify tumor response assessment for some patients during or after chemotherapy. The risk of PD associated with platinum/pemetrexed, the doublet with the lowest rate of early treatment discontinuation that was associated with no tumor response assessment, might be overestimated when compared with other platinum-based doublets. Second, our study could have underestimated clinical toxicity because of the lack of information on AEs that occurred outside of the four participating hospitals. Third, our study did not adjust prophylaxis treatment for non-hematological AEs and the true differences in non-hematological toxicity among the studied platinum-based doublets were unlikely observed in our study. Fourth, the study results are based on highly selected patients using the propensity score methods, which limit the external validity [28] of the findings, despite confirmation of internal validity by conventional regression methods. Also, the multiplicity was not adjusted to control the type I error of this study. Finally, our study was unable to collect survival data to assess treatment effects associated with the studied platinum-based doublets. The observed superior clinical effectiveness associated with pemetrexed treatment has to be carefully interpreted until the impact of these treatment effects on overall survival is fully clarified.

## Conclusions

This large real-world retrospective cohort study with Chinese patients supports previous studies showing improved disease control associated with platinum-based pemetrexed doublet when compared to platinum-based doublet with gemcitabine in first-line setting for advNS-NSCLC. This study also indicated that pemetrexed treatment was also associated with lower risk of treatment failure compared to the other third-general cytotoxic agents combined with platinum treatment in the first-line setting for advNS-NSCLC. The toxicity data suggest that pemetrexed, docetaxel, and paclitaxel-contained doublets had comparable toxicity profiles, but may be less toxic than gemcitabine or vinorelbine-contained doublet. Finally, pemetrexed treatment was associated with the lowest risk of early treatment discontinuation (versus all other doublets) and the shortest length of hospital stay among the treatments (versus paclitaxel, vinorelbine or gemcitabine doublets) in the first-line setting for advNS-NSCLC likely because of its superior effectiveness and less toxicity.

## Abbreviations

NSCLC: Non-small cell lung cancer
advNS-NSCLC: Advanced non-squamous NSCLC
GDP: Gross domestic product
CAMSTH: Chinese Academy of Medical Sciences Tumor Hospital
XWH: Xuanwu Hospital
HNPTH: Hunan Province Tumor Hospital
XYH: Xiangya Hospital
TKI: Tyrosine kinase inhibitor
EGFR: Epidermal growth factor receptor
ECOG: Eastern Cooperative Oncology Group
WBC: White blood cell
RECIST: Response Evaluation Criteria in Solid Tumors
AE: Adverse event
CTCAE: Common Terminology Criteria for Adverse Events
G-CSF: Granulocyte colony-stimulating factor
EPO: Erythropoietin
IL-11: Interleukin 11
TPO: Thrombopoietin
CR: Complete response
PR: Partial response
SD: Stable disease
PD: Progressive disease
RR: Rate ratio
OR: Odds ratio.
